# Influence of the Duration of Intravenous Drug Administration on Tumor Uptake

**DOI:** 10.3389/fonc.2013.00192

**Published:** 2013-07-25

**Authors:** Sylvain Fouliard, Marylore Chenel, Fabrizio Marcucci

**Affiliations:** ^1^Clinical Pharmacokinetics Department, Institut de Recherches Internationales Servier, Suresnes, France; ^2^Centro Nazionale di Epidemiologia, Sorveglianza e Promozione della Salute (CNESPS), Istituto Superiore di Sanita’ (ISS), Roma, Italy; ^3^Hepatology Association of Calabria (ACE), Reggio Calabria, Italy

**Keywords:** tumor, uptake, size, infusion, affinity

## Abstract

Enhancing tumor uptake of anticancer drugs is an important therapeutic goal, because insufficient drug accumulation is now considered to be an important reason for unresponsiveness or resistance to antitumor therapy. Based on a mechanistic tumor uptake model describing tumor exposure to molecules of different molecular size after bolus administration, we have investigated the influence of the duration of intravenous administration on tumor uptake. The model integrates empirical relationships between molecular size and drug disposition (capillary permeability, interstitial diffusivity, available volume fraction, and plasma clearance), together with a compartmental pharmacokinetics model and a drug/target binding model. Numerical simulations were performed using this model for protracted intravenous drug infusion, a common mode of administration of anticancer drugs. The impact of mode of administration on tumor uptake is described for a large range of molecules of different molecular size. Evaluation was performed not only for the maximal drug concentration achieved in the tumor, but also for the dynamic profile of drug concentration. It is shown that despite a lower maximal uptake for a given dose, infusion allows for a prolonged exposure of tumor tissues to both small- and large-sized molecules. Moreover, infusion may allow higher doses to be administered by reducing Cmax-linked toxicity, thereby achieving a similar maximal uptake compared to bolus administration.

## Introduction

Solid tumors are characterized by important abnormalities in tissue architecture and composition (1). These abnormalities represent considerable obstacles for uptake and penetration of antitumor drugs. Thus, tumor blood supply is often inefficient and, consequently, drug delivery to the tumor is impaired. Also the transvascular and interstitial transport of antitumor drugs is impaired because of reduced transvascular pressure gradient, high interstitial fluid pressure (2), high packing density of tumor cells (3), intercellular junctions (4), and altered composition of the extracellular matrix that increases frictional resistance (5). These abnormalities compromise the tumor delivery of antitumor drugs of all molecular sizes, i.e., low molecular weight drugs, macromolecular drugs, and nanoparticulate drug formulations. In fact, transvascular and interstitial transport of molecules is governed by flow (convection) and diffusion from regions of high concentration to regions of lower concentration. For macromolecules diffusion is extremely slow, and they are transported mainly by convection, that is, by streaming of a flowing fluid (6). As regards low molecular weight drugs, many of them show significant binding to plasma proteins, which leads them to behave, functionally, like macromolecules. Convection-driven transport, however, is often compromised in solid tumors because of decrease or loss of the transvascular pressure gradient.

Cytotoxic drugs (chemotherapeutics or antibodies mediating antibody-dependent cellular cytotoxicity or complement-dependent cytotoxicity) can, at least in part, limit the negative consequences of these effects. In fact, it has been proposed that cytotoxic effector drugs that are administered repeatedly at regular intervals cause “peeling” of increasing numbers of tumor cell layers until tumor regression is observed (2, 7, 8). Such a mechanism of action is expected to suffer less from the negative consequences of an impaired interstitial transport and penetration. The tumor cell layers that are eliminated are the most proximal to the tumor vessels from which the drug extravasates. Elimination of vessel-proximal tumor cell layers, however, may stimulate proliferation and repopulation of more vessel-distant tumor cells leading them to replace the cells that have been eliminated as a result of drug-induced cytotoxicity. This can be an important cause of treatment failure (9). Moreover, cytotoxic drugs can also promote active mechanisms of resistance induction. Thus, it has been shown that intermittent treatment of mice bearing ovarian cancer xenografts with docetaxel led to the development of different mechanisms of drug resistance, while continuous drug infusion resulted in superior antitumor efficacy and prevented drug resistance (10). These results suggested that continuous drug infusion may have considerable advantages over the more commonly used, intermittent, bolus administration protocols (11).

On the basis of these considerations it appeared of obvious interest to elaborate mechanistic models that describe the effects of continuous infusion on the tumor uptake of molecules compared to bolus administration. We have performed such a study taking advantage of a mechanistic tumor uptake model that had been described for bolus administration (12). In this report we describe the results of this study and compare them with those obtained for bolus administration.

## Materials and Methods

Simulations were performed using the equations of the model described by Schmidt and Wittrup (12), implemented in R (13) and modified in its pharmacokinetic components in order to integrate the intravenous administration rate. The model describes the relationships between molecular radius (*R*_mol_) and permeability across the tumor capillary wall (*P*), diffusivity within the tumor interstitium (*D*), available volume fraction in the tumor (ε), and rate of plasma clearance (*k*_clear_), respectively. These relationships are based on previously reported experimental measurements for molecules of various sizes in tumor tissues [supplementary data from Schmidt and Wittrup (12)].

The impact of molecular radius (*R*_mol_) on diffusivity and available volume fraction was described by modeling tumor tissue as a series of small and large right circular cylindric pores (14). Diffusivity of molecules in each pore (*D*_poretum_) can be estimated from diffusivity in solution (*D*_free_) and the ratio (λ) of molecular radius (*R*_mol_) to pore radius (*R*_pore_) using the equations:

$$\begin{array}{llll} D_{\text{poretum}} & =D_{\text{free}}\; & & \;\\ & \;\times \frac{1-2.105\lambda +2.0865\lambda^{3}-1.7068\lambda^{5}+0.72603\lambda^{6}}{1-0.78587\lambda^{5}}\; & & \;\\ D_{\text{free}} & =\frac{3\times \frac{10^{-6}\text{c}{\text{m}}^{2}}{\text{s}}}{R_{\text{mol}}}\; & & \; \end{array}$$

for λ < 0.6. For λ > 1, *D*_poretum_ = 0. For other values of λ, the ratio *D*_poretum_/*D*_free_ was determined from previously described work (15). *R*_mol_ is expressed in nanometers. Overall, diffusion within the tumor is:

$$D=A\times D_{\text{poretu}{\text{m}}_{\text{small}}}+B\times D_{\text{poretu}{\text{m}}_{\text{large}}}$$

where *A* and *B* are the relative diffusions occurring in small and large pores, respectively. According to this two-pore tumor model, the available volume fraction is defined as:

$$\varepsilon =V_{i}\left(A\times \varphi_{\text{poretu}{\text{m}}_{\text{small}}}+B\times \varphi_{\text{poretu}{\text{m}}_{\text{large}}}\right)$$

where *V*_i_ is the interstitial fluid volume fraction [approximated at 0.5 (16)], and partition coefficients for each pore size (φ_pore_) is (1 − λ^2^) when λ < 1, and 0 when λ > 1 (17). Vascular permeability was also modeled using a two-pore model of the capillary wall, and transport was assumed to be mainly diffusive; therefore, permeability across each pore was:

$$P_{\text{porecap}}=D_{\text{porecap}}\times \varphi_{\text{porecap}}$$

Overall, total permeability was defined as:

$$P=A_{\text{cap}}\times P_{\text{poreca}{\text{p}}_{\text{small}}}+B_{\text{cap}}\times P_{\text{poreca}{\text{p}}_{\text{large}}}$$

The impact of molecular size was modeled both on the renal plasma clearance (CL_R_) and the non-renal plasma clearance (CL_NR_). For non-renal plasma clearance, an empirical model accounted for loss of molecules above the cutoff size for glomerular filtration with an empirical model:

$$CL_{\text{NR}}=CL_{\text{NR,0}}-\delta \frac{R_{\text{mol}}}{R_{\text{mol}}+\gamma }$$

where CL_NR,0_ is the non-renal clearance for small molecule tracers (set to 2 mL/h), and δ (mL/h) and γ (nm) are empirical constants fit to the data. Renal plasma clearance is modeled as CLR = GFR × θ where GFR is the glomerular filtration rate (10 mL/h) and θ is the macromolecular sieving coefficient, depending on molecular size:

$$\theta =\frac{\phi K_{\text{conv}}}{1-{\text{e}}^{-\sigma P_{e}}+\phi K_{\text{conv}}{\text{e}}^{-\sigma P_{e}}}$$

where Φ is the equilibrium partition coefficient, σ is a correction term for the geometry of the glomerular slits approximately equal to 2 for baseline glomeruli, *K*_conv_ is the solute hindrance factor for convection, and *P*_e_ is the Péclet number defined as:

$$P_{e}=\frac{\phi K_{\text{conv}}\times v\times L}{\phi K_{\text{diff}}\times D_{\text{free}}}$$

where *v* is the fluid velocity vector (0.001 cm/s), *L* is the membrane thickness [100 nm in mice (18)], and *K*_diff_ is the diffusive hindrance factor. *K*_conv_ and *K*_diff_, along with the partition coefficient, are empirically modeled as (19)ϕ*K*_diff_= exp(−α*R*_mol_) and ϕ*K*_conv_= exp(−β*R*_mol_).

Plasma clearance (CL) was derived from renal and non-renal components CL = CL_R_ + CL_NR_, and along with plasma volume *V* (2 mL in mice), constituted the pharmacokinetic parameters of the one-compartment pharmacokinetic model.

Eventually, the tumor uptake was computed using a compartmental pharmacokinetic model in equilibrium with the tumor interstitium and a drug/receptor binding model. Considering Ω defined as:

$$\Omega =\left(\frac{2PR_{\text{cap}}}{\varepsilon R_{\text{Krogh}}^{2}}\right)\left(\frac{K_{d}}{\left(\left[A_{\text{g}}\right]∕\varepsilon \right)-K_{d}}\right)+K_{e}\left(\frac{\left(\left[A_{\text{g}}\right]∕\varepsilon \right)}{\left(\left[A_{\text{g}}\right]∕\varepsilon \right)-K_{d}}\right)$$

the concentration in tumor after a single bolus administration is:

$${\left[AB\right]}_{\text{tumar}}=\left(\frac{2PR_{\text{cap}}}{R_{\text{Krogh}}^{\text{2}}}\right)\left(\frac{\text{Dose}/V_{\text{plasma}}\left(e^{-k_{{\text{clear}}^{t}}}-e^{-\Omega t}\right)}{\left(\Omega -k_{\text{clear}}\right)}\right)$$

tumor concentration after intravenous infusion of rate, Rate = Dose/*T*_perf_, when *t* > *T*_perf_ is:

$$\begin{array}{l} {\left[AB\right]}_{\text{tumar}}=\int_{0}^{t}\left(\frac{2PR_{\text{cap}}}{R_{\text{krogh}}^{2}}\right)\\ \;\times \left(\frac{\text{Rate}/V_{\text{plasma}}\left(e^{-k_{{\text{clear}}^{\left(t-u\right)}}}-e^{-\Omega (t-u)}\right)}{\left(\Omega -K_{\text{clear}}\right)}\right)\text{du} \end{array}$$

which can be rewritten as:

$$\begin{array}{l} {\left[AB\right]}_{\text{tumar}}=\left(\frac{2PR_{\text{cap}}}{R_{\text{Krogh}}^{\text{2}}}\right)\\ \;\times \left(\frac{\text{Rate}/V_{\text{plasma}}}{\left(\Omega -K_{\text{clear}}\right)}\right)\left(\frac{1-e^{-k_{{\text{clear}}^{\text{t}}}}}{k_{\text{clear}}}-\frac{1-e^{-\Omega t}}{\Omega }\right) \end{array}$$

and tumor concentration after intravenous infusion of rate, Rate = Dose/*T*_perf_, when *t* < *T*_perf_ is:

$$\begin{array}{l} {\left[AB\right]}_{\text{tumar}}=\int_{0}^{T_{\text{perf}}}\left(\frac{2PR_{\text{cap}}}{R_{\text{krogh}}^{2}}\right)\\ \;\times \left(\frac{\text{Rate}/V_{\text{plasma}}\left(e^{-k_{{\text{clear}}^{\left(t-u\right)}}}-e^{-\Omega (t-u)}\right)}{\left(\Omega -K_{\text{clear}}\right)}\right)\text{du} \end{array}$$

which can be rewritten as:

$$\begin{array}{l} {\left[AB\right]}_{\text{tumar}}=\left(\frac{2PR_{\text{cap}}}{R_{\text{Krogh}}^{\text{2}}}\right)\left(\frac{\text{Rate}/V_{\text{plasma}}}{\left(\Omega -K_{\text{clear}}\right)}\right)\\ \;\times \left(\frac{e-k_{{\text{clear}}^{t}}\left(e^{k_{\text{clear}}\;T_{\text{perf}}}-1\right)}{k_{clear}}-\frac{e^{-\Omega t}\left(e^{\Omega T_{\text{perf}}}-1\right)}{\Omega }\right) \end{array}$$

where Dose is the amount of drug administered, *t* is the time, [Ag] is the target antigen concentration (mol/L), *k*_e_ is the rate of endocytic clearance (s^−1^), *K*_d_ is the affinity of the targeting molecule for the antigen (mol/L), *R*_cap_ is the capillary radius (μm), and *R*_Krogh_ is the average radius of tissue surrounding each blood vessel (μm).

Simulations of tumor uptake versus time profiles were performed for both intravenous bolus administration and continuous infusion. Duration of continuous infusion *T*_perf_ was set to 60 h with no loss of generality. The range of molecular radius (*R*_mol_) for simulations was set from 0.1 to 100 nm, and the corresponding molecular weight (MW, expressed in kDa) was approximated as $\text{MW }=\;1.32\times R_{\text{mol}}^{3}$. The range of affinity for the target (*K*_d_) for simulations was [10^−12^; 10^−6^] (*K*_d_ was set to 10^−9^ when investigating tumor uptake/time relationship). The case of IgG molecules is out of the scope of the present work, as their plasma clearance is smaller than other molecules with the same molecular weight due to their binding to FcRn receptors (20).

Simulations were performed using estimated parameter values described in Table 1, consistently with values used by Schmidt and Wittrup (12). In order to better assess differences in tumor uptake between modes of administration, simulations were performed both using the same administered dose (*D*) for bolus administration and continuous infusion, and using *D* and 100 × *D* for bolus administration and continuous infusion, respectively. Tumor uptake was expressed as a fraction of injected dose/gram (% ID/g).

**Table 1:** **Definition of the parameters and values used for simulations**.

Parameter	Definition	Value
MW	Molecular weight (kDa)	1–1000
*R*_tumsmall_	Radius of smaller tumor pore within tumor (nm)	13.8
*R*_tumlarge_	Radius of larger tumor pore within tumor (nm)	1000
*R*_capsmall_	Radius of smaller tumor pore within capillary wall (nm)	4.5
*R*_caplarge_	Radius of larger tumor pore within capillary wall (nm)	500
*A*_tum_	Partition coefficient in smaller pores within tumor (−)	0.9
*B*_tum_	Partition coefficient in larger pores within tumor (−)	0.1
*A*_cap_	Partition coefficient in smaller pores within capillary wall per unit membrane thickness (cm^−1^)	17.6
*B*_cap_	Partition coefficient in larger pores within capillary wall per unit membrane thickness (cm^−1^)	0.65
*V*_i_	Interstitial fluid volume fraction (−)	0.5
GFR	Glomerular filtration rate (mL/h)	10
α	Empirical fitting constant (nm^−1^)	1.6
β	Empirical fitting constant (nm^−1^)	0.95
γ	Empirical fitting constant (nm)	0.2
δ	Empirical fitting constant (mL/h)	1.94
*v*	Fluid velocity vector (cm/s)	0.001
*L*	Membrane thickness (nm)	100
CL_NR,0_	Non-renal clearance for small molecules tracers (mL/h)	2
V_plasma_	Plasma volume (mL)	2
σ	Correction term for geometry of glomeruli (−)	2
*R*_cap_	Capillary radius	8
*R*_Krogh_	Average radius of tissue surrounding blood vessels (μm)	75
*K*_d_	Molecule affinity for antigen (mol/L)	10^−12^–10^−6^
*K*_e_	Rate of endocytic clearance (1/s)	0.000016
[*A*_g_]	Target antigen concentration in the tumor (nmol/L)	1.5

## Results

We simulated the influence of the molecular radius, the time-course, and the affinity on the maximal tumor uptake of molecules administered by continuous infusion. Duration of infusion was set to 60 h.

**Figure 1:**
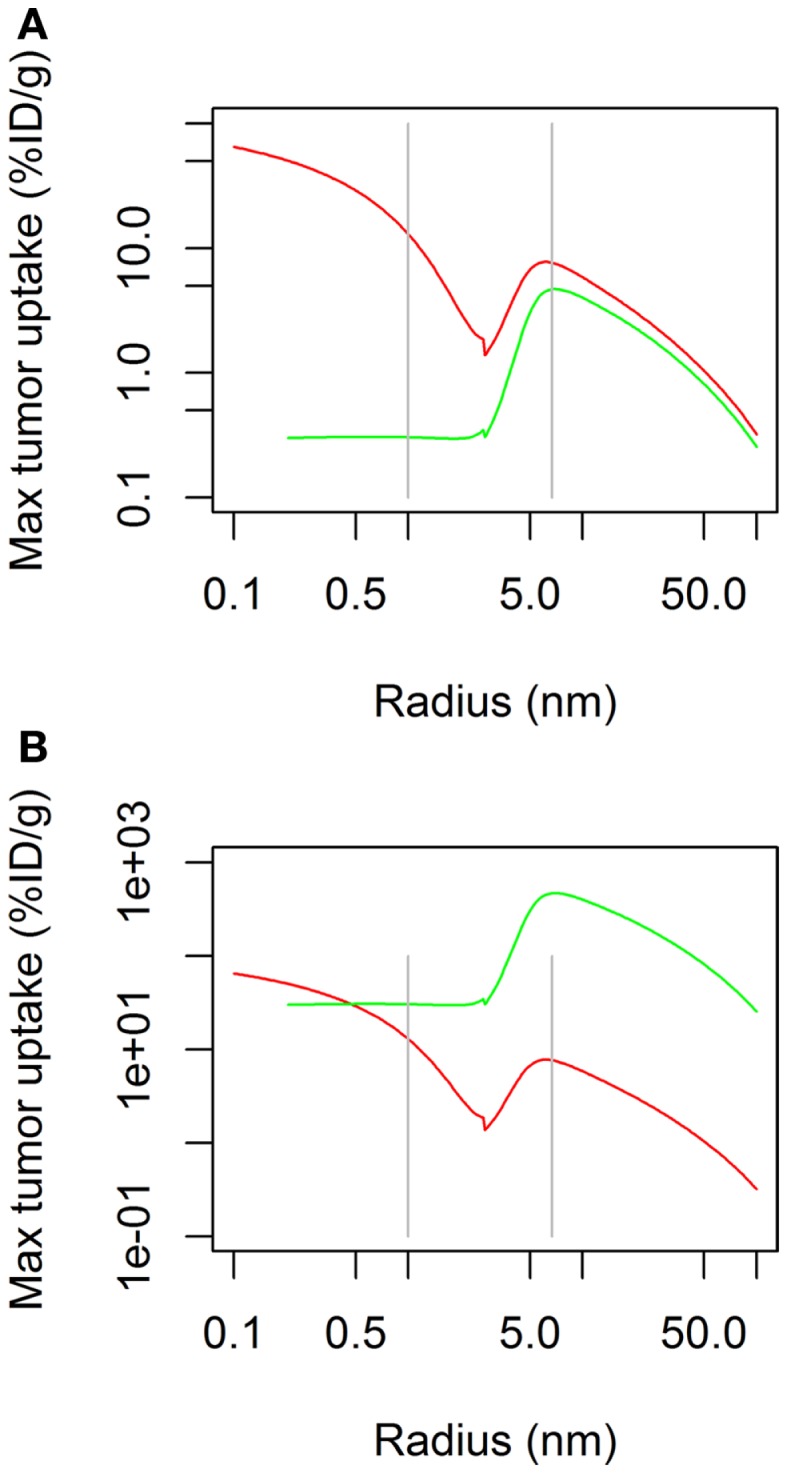
**Maximal tumor uptake as a function of molecular radius after continuous infusion (green) or bolus administration (red)**. Administered dose is the same for both modes of administration in **(A)**, but is 100× higher for continuous infusion in **(B)**.

The simulation of the influence of the molecular radius on maximal tumor uptake (ID/g) showed (Figure 1A) that maximal tumor uptake after continuous infusion was similar to that after bolus administration for large molecules (*R*_mol_ > 3 nm), but it was lower for small molecules (*R*_mol_ < 3 nm). The dose administered by continuous infusion was then increased by a factor of 100 in order to achieve a maximal tumor uptake (Figure 1B) for small molecules similar to that after bolus administration. Under these conditions, the reduced maximal tumor uptake of small molecules after continuous infusion was no longer observed. Regarding large molecules, the same pattern was observed after both modes of administration (i.e., bolus and continuous), with an increase to a maximal value of tumor uptake, and a decrease as molecules exceeded a size of ∼10 nm.

**Figure 2:**
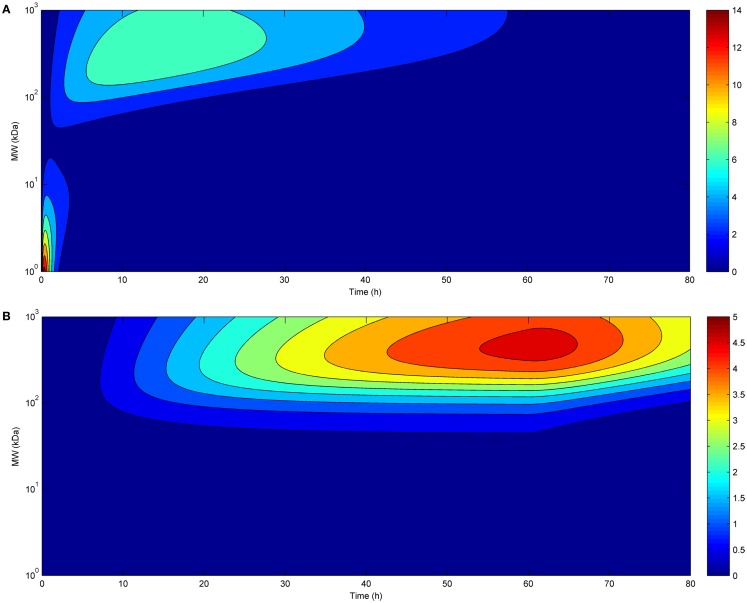
**Tumor uptake (color scale, in % ID/g) as a function of time (*x*-axis) and molecular radius (*y*-axis) after bolus administration (A) or continuous infusion (B) (same doses for both modes of administration)**.

The time-course of tumor uptake (Figures 2A,B) showed that the increase in concentration was delayed after continuous infusion compared to bolus administration, both for large and small molecules. However, peak tumor uptake of small molecules was more affected by the mode of administration than that of large molecules, i.e., the increase in tumor uptake was higher for small molecules than for large molecules. Regarding large molecules, while maximal tumor uptake was comparable between bolus administration and continuous infusion, tumor exposure was longer after continuous infusion. The benefit of a higher maximal tumor uptake of small molecules observed after bolus administration was balanced by the shorter duration of tumor exposure.

**Figure 3:**
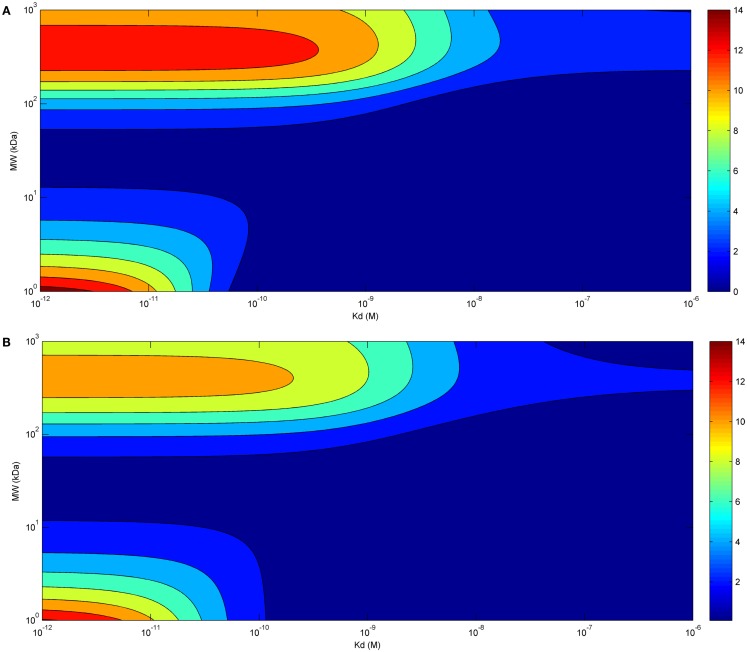
**Tumor uptake (color scale, in % ID/g) as a function of the affinity of the administered molecule for its receptor (*x*-axis) and molecular radius (*y*-axis), 24 h after bolus administration (A) and at the end of a 60 h-continuous infusion (B) (same doses for both modes of administration)**.

Eventually, we investigated the relationship between affinity and maximal tumor uptake (Figure 3). Increasing the affinity of a molecule increased its maximal tumor uptake up to a plateau value. This was seen for both bolus (Figure 3A) and continuous infusion (Figure 3B). The affinity at which this plateau value was attained depended for both modes of administration on the size of the administered molecule (10^−9^ for larger molecules, and 10^−11^ for smaller molecules). The fact that the affinity required to achieve a similar tumor uptake is much lower for larger than for smaller molecules shows that although the time-course of tumor uptake is strongly dependent on the mode of administration, the relationship between tumor uptake and affinity is unaffected.

## Discussion

In this article we have simulated the influence of molecular radius, time-course, and affinity of a molecule (e.g., an antitumor drug) on its maximal tumor uptake after continuous infusion and compared the results with those obtained in a similar model after bolus administration (12). For continuous infusion we set the duration to 60 h, a time period sufficient to attain equilibrium between the different compartments of the body.

We found that administration of a molecule by continuous infusion led to a relatively homogeneous uptake, that was independent of the molecular radius. A further increase of uptake, with a bell-shaped curve, was observed for molecules with a radius of ∼5–20 nm, with a maximal uptake at ∼10 nm. This is likely due to increased systemic accumulation of molecules that are larger than the size allowing for elimination through kidney filtration. Not surprisingly, at similar doses, maximal tumor uptake is much higher for bolus administration than continuous infusion, but this can be overcome by increasing the dose administered by continuous infusion (in Figure 1B, the dose administered by infusion is 100× higher than that administered by bolus). It is interesting to note that the shape of the uptake curve upon continuous infusion did not show the uptake minimum at ∼3 nm (25 kDa molecular weight) that is observed after bolus administration. This molecular size corresponds to that, for example, of a bispecific single-chain variable fragment. A compound of this kind (blinatumomab) is now in advanced clinical trials for the treatment of lymphoma acute lymphoblastic leukemia, and it is interesting to note that it is administered to patients by continuous infusion (21, 22). While lymphoma therapy is expected to suffer less from the impediments that characterize solid tumors, it appears, nonetheless, that administration of agents of this molecular size by continuous infusion is optimal to achieve the highest possible tumor uptake and accumulation. Overall, continuous infusion appears to be preferable to bolus administration in view of the possibility of achieving a more predictable tumor uptake of molecules of varying molecular size.

Also regarding the time-course of tumor uptake, continuous infusion appears to present advantages compared to bolus administration, allowing for longer exposure of the tumor. For small molecules, maximal tumor uptake was higher for bolus administration, but, again, this can be easily overcome by increasing the dose administered by infusion. Eventually, the relationship between tumor uptake and affinity of the administered molecules appears to be independent of the mode of administration. Thus, in accordance with previous results obtained with a similar model (12), the affinity required to achieve a similar tumor uptake is much lower for larger than for smaller molecules, and this is true for both bolus administration and continuous infusion.

Overall, the results from the mechanistic model used in this study suggest that continuous infusion offers some advantages compared with the more commonly used bolus administration. Most importantly, differences in uptake between molecules of different molecular size become less relevant upon continuous infusion than bolus administration. In particular, the nadir in tumor uptake at a ∼3 nm size disappears. Moreover, infusion allows for a prolonged exposure of tumor tissues to both small- and large-sized molecules. Eventually, this mode of administration may allow higher doses to be administered by reducing Cmax-linked toxicity, thereby allowing a similar maximal uptake compared to bolus administration. These advantages add to those related to reduced induction of drug resistance as a consequence of more homogeneous distribution of the drug throughout the tumor (10), thereby preventing or limiting repopulation of the tumor by proliferating tumor cells (9) and inhibiting induction of active mechanisms of resistance induction (7).

## Conflict of Interest Statement

The authors declare that the research was conducted in the absence of any commercial or financial relationships that could be construed as a potential conflict of interest.
